# Generalize or Personalize - Do Dogs Transfer an Acquired Rule to Novel Situations and Persons?

**DOI:** 10.1371/journal.pone.0102666

**Published:** 2014-07-16

**Authors:** Anne Hertel, Juliane Kaminski, Michael Tomasello

**Affiliations:** 1 Max Planck Institute for Evolutionary Anthropology, Department of Developmental and Comparative Psychology, Leipzig, Germany; 2 University of Bremen, Institute of Ecology, Bremen, Germany; 3 University of Portsmouth, King Henry Building, Portsmouth, United Kingdom; CNR, Italy

## Abstract

Recent studies have raised the question of whether dogs, like human infants, comprehend an established rule as generalizable, normative knowledge or rather as episodic information, existing only in the immediate situation. In the current study we tested whether dogs disobeyed a prohibition to take a treat (i) in the presence of the communicator of the ban, (ii) after a temporary absence of the communicator, and (iii) in the presence of a novel person. Dogs disobeyed the rule significantly more often when the communicator left the room for a moment or when they were faced with a new person, than when she stayed present in the room. These results indicate that dogs “forget” a rule as soon as the immediate human context becomes disrupted.

## Introduction

In the past two decades, the domestic dog (*Canis lupus familiaris*) has become increasingly popular as a model organism for comparing cognitive abilities across species boundaries. This is because dogs are especially skilled at understanding communicative signals given by humans. Considering the multiple ways dogs work and interact with us, understanding human signals should be highly adaptive [1]. Comparing the pathways through which information is internalized in dogs and human infants is a crucial approach which targets the very roots of human culture. Learning is characterized as the “generalization of the originally acquired information: to new occasions, new locations, new objects, new contexts, etc” ([2], p. 148). Human infants start to imitate instrumental acts at as early as nine months, indicating that a demonstration is understood as teaching of a conventional normative practice [3]. 2- to 3-year-olds quickly learn a rule in a new game and comprehend this rule as normative [4]. Actions that follow conventional norms are applicable to everybody, are context-specific [5] and other persons are corrected when applying a different technique [4], [6]. From three years onwards children relate a learned rule to the specific context and know when protest at rule violation is applicable [4], [7]. At four years of age, children discriminate between demonstrators and selectively learn from knowledgeable, familiar and reliable persons [8]. This behaviour –assigning a specific use and function to an object based on imitation of a knowledgeable informant, to expect others to use it the same way and to comprehend a deviation from the normative standard or conventional use as a mistake – is the basis of human culture. One of the most interesting questions in this regard is how children know that a piece of information is universally applicable and thus generalize it. The concept of natural pedagogy implies that children are receptive to learning from others when being addressed in an ostensive- referential way [2]. This includes signals like eye contact, gaze alternation between child and the referential object, addressing the child by name or speaking in a high-pitched voice [9]. This pathway of transmitting generic knowledge by communicating rules seems to be a uniquely human phenomenon. Animals on the other hand have been shown to truly learn only through observation and association [10], but can apply a communicative transmission pathway to transfer a piece of episodic information which is relevant to the current situation only [2]. Linking these two pathways and passing on generalizable knowledge through communication has yet not been shown for any animal species [2]. This study aims to explore the extent to which the domestic dog is capable of rule-mediated learning, allowing it to comprehend a given piece of information as a conventional norm and thus as generic knowledge. Because dogs are especially sensitive and skilled to understand human given ostensive cues one would expect that they might be particularly suitable for the concept of natural pedagogy and thus for rule mediated learning. This learning pathway would also be especially beneficial for dogs, as their primary living companions are humans. Generalization of knowledge would equip them with a faster learning pathway.

A recent study by Topal and colleagues [11] suggested that dogs are not able to transfer a rule from its communicator to a novel person. However, they proposed that instead dogs anchor a piece of information to its communicator. Dogs would thus not comprehend a communicated rule as universally applicable and would not be capable of normative learning. The authors tested their approach with the use of a common paradigm of Piagetian developmental stage theory [12], the A-not-B error. After having witnessed an object disappearing repeatedly behind a location (A) and having successfully retrieved it, one-year-old children fail to switch to another location (B) after having witnessed the object disappearing behind the first one. Instead they commit a perseverative search error and keep searching at location A. This paradigm is particularly suitable for testing the effect of communication on learning, as it has been shown that children commit the error significantly more often when it is presented in a communicative way than in a non-communicative or non-social context [13]. Communicative signals while hiding the object repeatedly at location A apparently mislead infants, causing them to understand the demonstration as a teaching lesson and thus the given information as being normative (“this object is always to be found at location A”) rather than episodic knowledge (“this object is now to be found at location A”). Similarly, adult pet dogs were more prone to perseverate when faced with a communicative presentation of the A-not-B task, and the occurrence of the error was substantially reduced when the hiding was presented in a non-communicative or non-social context [11]. These results suggest that, like in children, communicative cues have a decisive effect on the dogs’ responses. However, whether dogs – like human infants – actually interpret the A trials as teaching lessons and comprehend the location of the toy behind screen A as the norm is debatable. To test this, the presenter of the A trials – and thus the communicator of the rule – was replaced by a novel person in the B trials. Children continued to erroneously choose location A when B trials were presented by a novel person [11]. They were therefore generalizing the knowledge to the new situation. Dogs on the other hand searched significantly more often at the correct B location when faced with the new person. Hence dogs apparently did not perceive what they witnessed during demonstrations as being universally applicable. The authors concluded that dogs associate a given piece of information with the person who communicated it. Infants on the other hand readily transferred the information to the new experimenter. This supports the notion that communicating generalizable information is a uniquely human learning path [2], [14].

According to this study, the presence of different persons has different effects on the dogs’ responses. It does however remain unclear to what extent the interruption of the situation itself, such as leaving the room and returning, affects the observed outcome. Generally, the presence of a demonstrator plays a crucial role in modulating a dog’s response. It has also been shown that dogs alter their behaviour according to the attentional state of a human [15], [16]. After having witnessed a human forbidding them from taking a piece of food, dogs obeyed the ban when the human was present; if however the person left the room, they ignored or forgot the rule and took the treat. Furthermore, dogs took the food more often when the person was distracted or turned her back to the dog than when she watched the dog. Topal et al. [11] nicely showed that switching people resulted in a switch of behaviour. The authors interpreted this as an indication that dogs do not generalize the observed rule to a new person but instead anchor it to the person presenting the demonstration trials. We argue that there is one aspect which was not controlled for in the study design which could explain the results obtained. This is that leaving the room and coming back itself could reduce the probability of committing the error. If this were true, not committing the error in the presence of a second person could not only be explained by personalization of a rule to the demonstrating person. In fact this would indicate that an acquired rule is comprehended as truly episodic knowledge and loses relevance as soon as another action like for example leaving the room interrupts the situation.

To investigate this idea we tested dogs in a prohibitive setup. Although it is not possible to test whether dogs understand a rule as a normative convention by measuring the amount of intervention behaviour, as is common practice in research with children, we still based our study design on one used by Rakoczy et al. [7]. In this study a rule labelled an action with an object as wrong at one location and as correct at another. Three-year-olds were able to discriminate between the location at which a rule is effective and one at which it is not. In our study there were two possible locations to obtain a reward; one was more favourable than the other one since it was closer to the dog. First we tested how prone dogs were to disobey a rule in the presence of a person who established it. The rule was that it is forbidden to take the favourable nearby location and it was established during demonstration trials. In this control treatment dogs were expected to show that they are in fact able to accept a rule communicated by a demonstrator. In addition we tested whether dogs would show the same response pattern when this person left the room for a brief moment and returned before the dog was allowed to choose and whether dogs would transfer the rule to a novel person. If dogs anchor a piece of information to the communicator we would expect them to choose the preferred location significantly less often when the experimenter stays in the room than when experimenters change. We would also expect dogs to choose the nearby location and thus disobey significantly less often when the same experimenter leaves the room and returns a moment later than when persons change. However, if dogs choose the preferred nearby location more often in this treatment than when the experimenter stays, this would indicate that the leaving process itself has a negative effect on the dogs’ memory of the rule.

## Methods

### Ethics Statement

No special permission for use of animals (dogs) in such socio-cognitive studies is required in Germany, wherefore IRB approval was not necessary. All procedures were performed in full accordance with German legal regulations and the guidelines for the treatments of animals in behavioral research and teaching of the Association for the Study of Animal Behavior (ASAB) [17]. Dog owners with their dogs participated on a volunteer basis.

### Subjects

In total 47 dogs (*Canis lupus familiaris*) of various ages and breeds were tested. All subjects lived as pets with their owners in Leipzig, a medium-sized German city. Dogs were recruited from the database of the Max Planck Institute for Evolutionary Anthropology, where their owners had registered and volunteered them for testing. Most dogs had taken part in various studies before but were unfamiliar with the procedures used in this study. Testing took place between 25 June and 7 September 2012. Five dogs had to be excluded from the experiment: four dogs were distracted by the separation from their owner or afraid of the Plexiglas door; one dog was deaf and we decided that he might not perceive the ostensive cueing in the same way as other dogs. Data from 42 dogs were included in an initial analysis, whereupon three more dogs were excluded because they did not display an unambiguous choice behaviour. Thus, data of 39 dogs- 19 males and 20 females- were included in the final analysis (see Table S1). The age of these dogs ranged between 8 months and 13 years (mean ± SE 5.69±0.52 yrs.).

### Facilities

All tests for this study took place in a room at the Max Planck Institute for Evolutionary Anthropology in Leipzig. The room was empty except for a video camera and the testing equipment, which included a Plexiglas fence 120 cm in height which served as a retaining device. Dogs were able to observe all actions of the experimenter. There was a door in the middle of the fence which could be opened by a magnetic mechanism from outside of the room.

### Experimental setup

**Figure 1:**
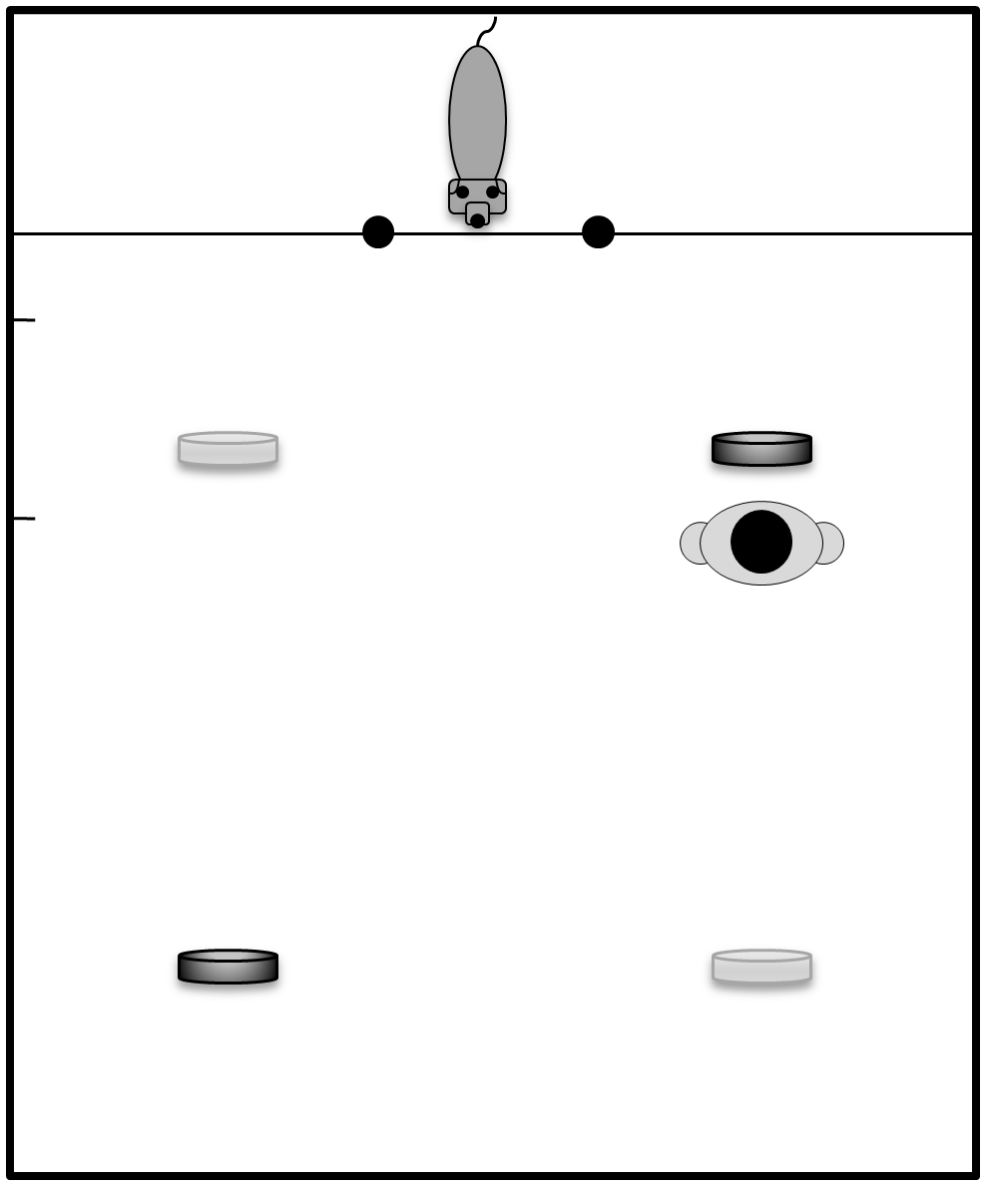
Arrangement of the experimental setup. From the doǵs perspective, the two dishes were placed diagonally either (i.) nearby to the right and distant to the left or (ii.) nearby to the left and distant to the right. After baiting, the experimenter positioned herself behind the nearby dish. Dogs were kept behind a translucent retaining device during baiting and rule establishment.

Two open plastic dishes (19×13.5×2.5 cm) were placed on the floor. One dish was placed 120 cm and the other 350 cm from the Plexiglas door (Figure 1). The distribution of distant and nearby dishes was diagonal, with the nearby one on the left and the distant one on the right side, or vice versa. Placements on the left- or right-hand side were randomized and counterbalanced across trials within treatments.

### Procedures

The general idea of the study was to see whether dogs would transfer a rule set up by one person (experimenter 1; E1) to a second person (experimenter 2; E2) if following the rule would mean rejecting a preferred solution. Therefore we used a setup based on prohibition. Dogs became familiar with the setting during pre-training. This was followed by four Preference trials (PT) to see which location (nearby vs. distant) dogs naturally preferred. The rule that it was forbidden to take a treat from the nearby location was established during demonstration trials and repeated before every experimental trial to avoid ceiling effects. Experimental treatments were presented in three distinct blocks. The sequence of these treatments was randomized and counterbalanced across all dogs to control for learning effects (Table 1, Table S1). Each treatment consisted of four trials. In the Stay treatment, E1 was present during the whole time until the dog chose a location; in the Return treatment she left the room for a brief moment before she came back and the dog was allowed to choose; and in the Switch treatment she left and E2 entered the room, in whose presence the dog was able to choose. Between the conditions dogs had a break of 5 minutes. In total, each dog received 4 Preference, and 12 pairs of demonstration and experimental trials. All trials were videotaped.

**Table 1:** Sequences in which experimental treatments were presented and respective Order ID; Preference tests (PT) were always carried out prior to the experimental trials.

Order ID	pos0	pos1	pos2	pos3
1	PT	Stay	Return	Switch
2	PT	Stay	Switch	Return
3	PT	Return	Stay	Switch
4	PT	Return	Switch	Stay
5	PT	Switch	Stay	Return
6	PT	Switch	Return	Stay

#### Pre-training

In the pre-training dogs were familiarized with all four locations from where food could be obtained (left nearby/distant; right nearby/distant) and also learned to walk through the Plexiglas door comfortably.

#### Baiting procedure

Regardless of the diagonal arrangement, baiting always started on the left-hand side from the experimenter’s perspective. The experimenter stood in front of the dog, showing it the treat and then walked to the location on the left side. Standing behind the dish, she called the name of the dog and said “Watch it!”. Then, while alternating her gaze between the dog and the dish she placed the treat in the dish, before walking to the location on the right side and repeating the procedure. Baiting was conducted by E1 in all experimental trials.

#### Preference test

The second experimenter baited both locations. She then left the room without giving any positive or negative cues and the Plexiglas door was opened. The dog could choose freely between the two locations without interference. After having chosen one location the dog was brought back behind the fence. A choice was defined as the dog touching the dish with its muzzle or paw and eating the treat. If a dog did not choose within 30 seconds, the trial was classified as ‘no choice’. Dogs were considered not motivated enough if they chose less than three times.

#### Demonstration trials

Demonstrations served to establish the rule that “it is forbidden to take food from the nearby location”. E1 therefore claimed ownership of the nearby dish by standing behind it and banning it with ostensive communication. She bent over the dish, guarding it with her hands and saying “This is mine!” with a firm voice, accompanied by gaze alternation. Then she stood straight and looked down at the dish. The Plexiglas door opened and the dog was allowed to roam freely through the room. Every approach towards the forbidden dish prompted a sharp “This is mine!” and if necessary was hindered by physically guarding the food. If however the dog decided to take the distant dish, no cueing or interference took place. Demonstration phases lasted 30 seconds and were repeated before every experimental trial. Placement of the dishes in the demonstration trials was always the same as in the following trial.

#### Experimental trials

After the baiting process, E1 stood behind the nearby dish, prohibiting it by saying “This is mine!”. Dogs were allowed to make one choice only. There were three treatments:

**Stay:** E1 kept standing behind the forbidden location, looking down at it. After 8 seconds the Plexiglas door opened and the dog was allowed to choose one location.
**Return:** After having expressed the rule, E1 left the room and returned after 5 seconds without looking at the dog. She stood behind her initial position and looked down at it. The Plexiglas door opened and the dog was allowed to choose.
**Switch:** E1 left the room after having expressed the rule. After 5 seconds E2 entered and took over her position without giving any communicative cues. The Plexiglas door opened and the dog was allowed to choose in E2’s presence.

In the experimental trials dogs were always allowed to take the treat from the location they chose first. There was neither positive nor negative cueing when the dog made its choice. The trial was over after the dogs made a choice or 30 seconds had elapsed.

### Coding

For preference and experimental trials we coded whether dogs took the nearby dish, the distant one or none at all. We also noted whether the choice made was for the left- or right-hand side. A dog’s response was scored as “disobey” when it took the nearby dish. 20% of all trials were re-coded by a person unfamiliar with the study for reliability analysis. Inter-rater reliability was assessed using a weighted Cohen’s Kappa coefficient with squared weights of differences. Reliability between the two coders was excellent (Cohen’s Kappa = 0.998, N = 181, *P*<0.001).

### Data analysis

All statistical analyses were computed in R [18]. Initial inspection of data for dogs as a group was used to exclude dogs which displayed an extensive “no choice” behaviour. This was defined as not choosing a dish in all four trials of two or more treatments. Inspection of residuals and QQ plots revealed that the data were not normally distributed. A Generalized Linear Mixed Model (GLMM, [19]) was applied using the function lmer of the R package lme4 [20]. Dispersion parameters of all models were examined by dividing the squared sum of the model’s residuals by the residuals’ degrees of freedom [21]. None of the implemented models were overdispersed. It was analysed if dogs were more prone to display a no choice behaviour in one of the three experimental treatments, at a specific point during the experiment (position 1–3) and the interaction between these two predictors. Dogs received four trials per treatment, wherefore the response variable ‘no choice’ was distributed between 0 and 4. A binomial distribution with logit link function was assumed. Holm- Bonferroni pairwise comparisons were used to compare treatments.

Following, the dogs’ proneness to disobey the rule was analysed. Data were not normally distributed and a GLMM was used. The response variable disobey was always distributed between 0 and 4, for which a binomial error distribution with a logit link function was assumed. The rate to disobey was analysed with regard to treatment, age and sex as fixed effects. Age was z-transformed to a mean of zero and a standard deviation of one. Likelihood ratio tests (R function ANOVA with argument test set to “Chisq”) were used for model optimisation. *P* values of the optimal model were Holm- Bonferroni adjusted [22] for pairwise comparisons of the performance between treatments. The significance level for the corrected *P* values was taken to be 0.05. Exact Wilcoxon signed rank tests were applied to all four treatments to compare the proneness to disobey for dogs as a group to chance probability using the package exactRankTests [23]. To account for the possibility of learning and position effects we ran a model with an interaction term between the three experimental treatments and the position in which they were received as a factor. Because the Preference test was always received in position 0, whereas the other treatments were randomly assigned to positions 1–3, the preference test had to be excluded from this analysis. Pairwise comparisons of the performance within a treatment, between the three possible positions, were implemented and *P* values were Holm-Bonferroni adjusted. To visualize these effects, estimates and standard errors from the summary output of the GLMM were back-transformed and multiplied by four, the actual number of trials. This was because in a binomial distribution data are proportionally distributed between 0 and 1. Possible side preferences were examined using a mixed model with binomial distribution and logit link function. Therefore, the total number of choices to the left- and right-hand side was pooled for each subject and the effect of side on the probability of taking a dish evaluated.

## Results

### Analysis of no choice behavior

**Figure 2:**
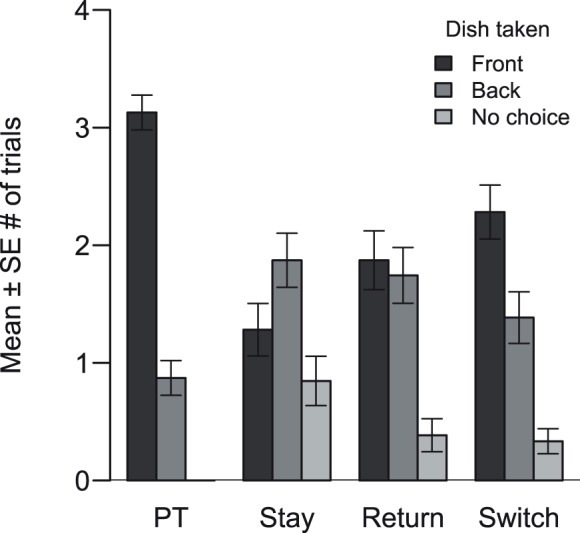
Choice behaviour for dogs as a group. Mean (±SE) number of trials in which dogs chose the nearby dish and thus disobeyed the ban, chose the hind dish or did not choose (N = 39). Choices are displayed for the Preference test (PT), when the experimenter stayed (Stay), left and returned (Return) or experimenters switched (Switch).

After a first data examination, three dogs were excluded from further analysis because they did not choose a dish in all four trials of two or more treatments. Although this behavior is in line with obeying the rule we could not exclude the possibility that dogs either did not understand the matter of the experiment or were too intimidated to choose. Results for the 39 remaining dogs are depicted in Figure 2. Out of 156 executed trials, dogs did not choose a dish in 61 trials, which is roughly 40%. In 20 cases a dog did not choose in one out of the four trials. In 5 cases two or three trials were no choice and in 4 cases a dog did not choose a dish in all four trials of one treatment. A mixed model including treatment as an explanatory variable was superior over a reduced model (Χ^2^ = 10.078, df = 1, *P* = 0.003), therefore treatment had a significant effect on the probability that a dog displayed a no choice behavior. Also position had a significant effect (estimate = −0.632, SE = 0.206, z = −3.059, *P* = 0.002). The negative estimate indicates that dogs were less likely to display a no choice response at the end of the experiment than at the beginning. Posthoc pairwise comparisons revealed that dogs were significantly more prone not to choose when the experimenter stayed in the room than when experimenters switched (*P* = 0.002). They were also less prone not to choose when the experimenter left and returned than when she stayed (*P* = 0.012). There were no significant differences in the probability not to choose a dish between the Return and the Switch treatment (*P* = 0.607). No significant effect of the interaction between treatment and position on the probability not to choose was found (Χ^2^ = 4.626, df = 2, *P* = 0.099).

### Treatment effects on a doǵs probability to disobey

**Figure 3:**
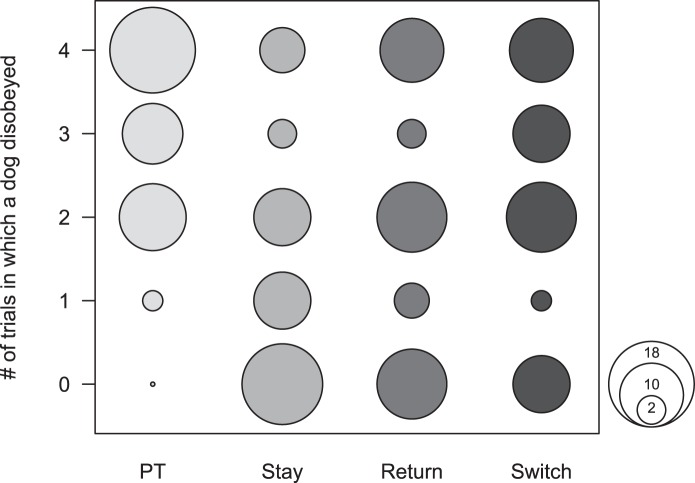
Variability of the dogś proneness to disobey in the four treatment situations. Displayed are the proportions of dogs that disobeyed the rule 0, 1, 2, 3 or 4 out of four received trials. Results are displayed separately for the Preference test (PT), when the experimenter stayed (Stay), left and returned (Return) or experimenters switched (Switch). N = 39.

A GLMM revealed no significant effect of the sex of a dog on its probability to disobey (*z* = 1.429, *P* = 0.153). Age however did have an effect. With increasing age dogs were more prone to choose the forbidden dish (estimate = 0.532, SE = 0.229, z = 2.325, *P* = 0.02). Treatment had a highly significant effect on the dogs’ responses, indicating that the probability to disobey the rule varied with the testing situation (Figure 3). A model including age and treatment revealed a superior fit over a reduced model without treatment (Χ^2^ = 96.187, df = 3, *P*<0.001).

Post-hoc pairwise comparisons between treatments revealed significant effects for all treatment comparisons. The probability to take the nearby dish was significantly lower in all three experimental treatments compared to the Preference test (PT - Stay: *P*<0.001, PT - Return: *P*<0.001, PT - Switch: *P*<0.001). Dogs disobeyed the ban significantly less often when E1 stayed in the room than when she left for a moment and returned (Stay – Return: *P* = 0.003) and or when experimenters switched (Stay – Switch: *P*<0.001). Also, the dogs took the nearby dish significantly more often and thus disobeyed the rule when the experimenter was replaced by another person than when the same person returned (Return – Switch: *P* = 0.031) (Table 2).

**Table 2:** Pairwise comparisons of the probability to disobey between experimental treatments.

	PT	Stay	Return
Stay	<0.001***	-	-
Return	<0.001***	0.003**	-
Switch	<0.001***	<0.001***	0.033*

*P* values of the between Treatment Post- hoc pairwise comparisons were Holm- Bonferroni adjusted; a significance level of 0.05 is assumed.

Performances of dogs as a group in each of the four treatments were compared against chance probability using Wilcoxon signed rank test. 69% of dogs chose the nearby dish three or four times during the Preference test, while 28% chose it in two trials and 3% only once (Figure 3). Therefore dogs as a group showed a preference for the nearby dish significantly above chance probability (Wilcoxon signed rank test: Tplus = 400.5, N = 28, *P*<0.001; mean correct 3.128). When confronted with the rule not to take the preferred nearby dish and in presence of the rule communicator, dogs chose the nearby dish and thus disobeyed significantly below chance level (Tplus = 380, N = 31, *P* = 0.006; mean correct 1.282). Now, only 17% of dogs took the nearby dish either 3 or 4 times, while 19% disobeyed twice and 62% only once or not at all. In both the Return and Switch treatments this effect vanished and dogs chose the nearby dish at chance level (Return: Tplus = 207, N = 27, *P* = 0.673; mean correct = 1.872, Switch: Tplus = 225, N = 27, *P* = 0.366; mean correct = 2.282). However, in the Return 31% of dogs chose the nearby dish three or four times, while 31% performed at chance and 38% chose it once or not at all. By contrast, in the Switch treatment 46% of dogs chose the nearby dish three or four times, 31% twice and 23% once or not at all (Figure 3).

### Order effects on the probability to disobey

**Figure 4:**
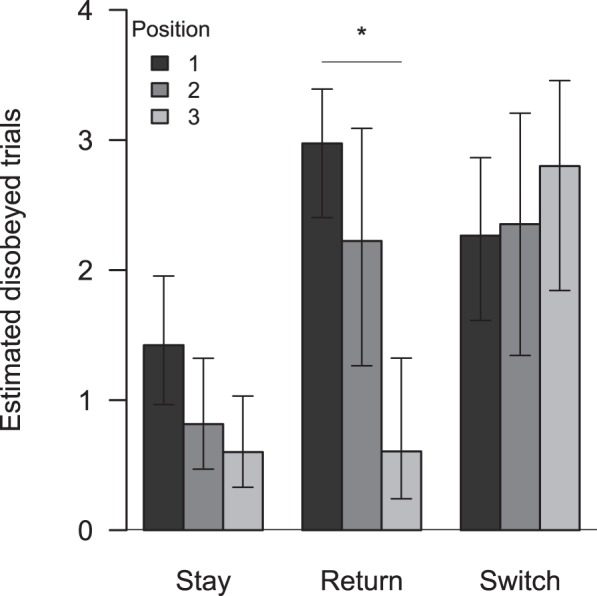
Effect of position in which each treatment was received on the probability to disobey the rule. Bars are based on back transformed estimates and SE of the corresponding GLMM on order effects and are therefore corrected for inter-individual variation. Displayed are estimated (±SE) number of trials in which dogs disobeyed the rule for the three experimental treatments with respect to position (1–3) in which the treatment was received. Each dog received all three treatments but each only in one position. N_per treatment_ = 39.

We found a significant effect of the position in which a treatment was received on the dogś probability to disobey. A model including the interaction term between treatment and position was superior over a reduced model without the interaction (X^2^ = 11.148, df = 4, *P* = 0.025; Figure 4). Bonferroni-corrected post-hoc pairwise comparisons revealed that the probability to disobey did not differ significantly between the three possible positions in the Stay and Switch treatments. Thus the effect was solely based on the Return treatment. When E1 left the room and returned, dogs disobeyed significantly more often when this was their first experience than when they received this treatment last (*P*<0.001). They also disobeyed more often, although not significantly so, when the treatment was received first compared to second (*P* = 0.071) or second compared to last (*P* = 0.071). Experience therefore increased the probability not to disobey significantly in this treatment. Sex (*z* = −1.164 *P* = 0.245) and age (*z* = −1.053, *P* = 0.292) did not have a significant effect on the probability to disobey in this model and where thus stepwise eliminated.

### Side effects

Dishes were distributed in a counterbalanced way to the left and right in all treatments, meaning no differences were expected in terms of dishes being taken on the left or the right – regardless of their being placed closer or farther away. Nevertheless, side had a significant effect on the probability of taking a dish. Dogs took a dish placed on the right side significantly more often than one on the left side (β = 1.25, SE = 0.139, *z* = −9.008, *P*<0.001).

## Discussion

In our experiment we confronted dogs with a rule and tested how prone they were to disobey it in the presence of the communicator of the rule, after an interruption of the situation and in the presence of a novel person. When a preferred choice was prohibited most dogs did not disobey this rule if the person who expressed the rule stayed present in the room whereas when experimenters switched, they disobeyed significantly more often. The rule was however not erased entirely, as dogs chose the nearby dish still significantly less often than when they had the free choice. These results are in line with an earlier study on generalization in dogs [11]. We agree with Topal et al. that the overall similarity of the test situation carries enough cues to trigger an intermediate response pattern. Dogs therefore do generalize what they have learned during demonstrations to some extent to the new situation or person. Our main concern however was to examine the true effect of the experimenters’ personality and the leaving process itself on the generalization of a rule. We proposed that a communicated rule might not so much be anchored to its communicator but that the information provided is in fact comprehended as existing only in the specific situation. The results of our study support this hypothesis to some extent. When the situation was interrupted, i.e. the demonstrating person left the room and returned a moment later, the dogs’ performance was intermediate to their performance in the Stay and Switch treatments and differed significantly from both of them. This is an indication that the personality of the experimenter is not the sole factor for disobeying the rule. We conclude that dogs were prone to reject the rule and disobey as soon as the situation was interrupted by the rule communicator’s leaving. Dogs disobeyed significantly less often when the rule communicator returned compared to when a new person returned. We attribute this effect to learning by repetition. We found a significant interaction between position and experimental treatment. This effect was limited to the Return treatment. The later this treatment was received, the less prone dogs were to disobey the rule. Thus the link between the rule and its communicator possibly became stronger in the course of the experiment. The response pattern observed for dogs as a group in the Stay and Switch treatments was not affected by presentational order and therefore irrespective of repetitive learning. We therefore furthermore conclude that the rule not to take the nearby dish was understood by the dogs right from the beginning, as we did not find any position effects in these treatments. In the Return treatment on the other hand, the dogs’ responses indeed depended on the number of conditions received so far. For inexperienced dogs, the leaving process apparently weakened the importance of the prohibition that had just been established and most of them disobeyed the rule by choosing the preferred nearby dish. Additionally, the repositioning of E1 behind the close location could have had an erroneous attracting effect. Although the communicative intent was a negative one, the nearby dish could have been made more salient through local enhancement [24]. Thus inexperienced dogs might consider the rule as less important when its communicator leaves the room or misinterpret the posture of the returning person as local enhancement and thus a new imperative upon which to act. The same mechanisms could be responsible for the dogś response pattern in the Switch treatment with the difference that here performance did not improve over time. The later the Return treatment was received, the less prone dogs were to disobey and the performance of subjects improved over time. We therefore conclude that dogs do not spontaneously anchor a rule to its communicator but that they will do so based on learning by repetition. As we did not focus on learning by repetition but on spontaneous rule mediated learning, position effects must be taken into account when interpreting the results of the Return treatment.

One problem of our study was that dogs did not choose a dish in 40% of all trials. Not choosing is in line with obeying the rule because dogs did not decide on the nearby dish. Still it cannot be ruled out that dogs might have been too intimidated by the prohibition to choose. Not choosing also occurred significantly more often in the Stay treatment compared to the Return or Switch treatments. This is also what we expected as the prohibition should be strongest in presence of its communicator. We excluded three dogs from further analysis because they displayed an extensive no choice behaviour and it was not clear if they understood the purpose of our experiment. Also dogs were more prone not to choose a dish at the beginning of the experiment compared to the end. This could be because dogs were surprised by the prohibition and forgot about the existence of the hind dish. It is also possible that they were more intimidated when the rule was just established. By analysing the dogś proneness to disobey a rule, which was an unambiguous choice of the nearby dish, we avoid potential misinterpretation of the no choice trials.

Placement of dishes was counterbalanced to the left and right, still the side on which a dish was placed did have an effect on the probability of being chosen. Dogs were more prone to select a dish on the right hand side. There is evidence from the literature that children understand a location as an episodic feature which can change rather than a generalizable one [2]. Contrary to this, Rakozcy et al. [7] used location as a tool to test whether children understand context-relativity of a rule. In fact three-year-olds successfully discriminated between where a rule applied and where it did not. Also Dumas [25] and Ashton and De Lillo [26] showed that for dogs, location is an extremely salient cue. In the study at hand, side had an effect which however did not override treatment effects.

Earlier studies have shown that dogs are sensitive to the attentional state of humans [15], [16]. The study presented here is the first to evaluate dogs’ reactions to temporary inattentiveness of humans. Our results show that dogs revoked a rule as soon as the communicator was temporarily absent and inattentive. They only anchored it to the communicator after extensive repetitions. It remains unclear whether an interruption made the dogs regard the rule about not taking the nearby dish as invalid or whether they misinterpreted the posture of the returning person behind this dish as local enhancement and therefore a new imperative upon which to act. Dogs were prone to reject the rule as soon as the immediate situation was disrupted, and even more so when the rule communicator was substituted by a novel person. This is consistent with the findings of Topal et al. [11].

Although through domestication dogs have evolved unique skills to understand and communicate with humans [27], [28], transferring conventional knowledge through communication rather than association or observation still seems to be a uniquely human strategy [2], [26]. An earlier study showed that dogs regard a rule as being valid in the presence of its communicator but do not generalize it to novel persons [11]. Based on the results of our study we object to the conclusion of Topal et al. [11] that dogs associate a rule with the communicator and likewise suggest that they attach it to the immediate situation. We conclude that dogs are not able to learn through communicating rules. This is different from young infants, who readily conceive communicated information as conventional and transferable to new contexts and persons, which can be derived from the fact that they correct others who apply an approach that differs from the convention [2], [4], [5]. In line with these findings, Ashton and De Lillo [26] found rudiments that dogs can comprehend communicated information and rule mediated learning in a spatial search task to some extent but this could not replace associatively learned knowledge. The study at hand adds further insight into the processes of learning in dogs.

## Supporting Information

Table S1 **List of all dogs included in the analysis.** Information about sex, age, breed and the order in which a dog was tested are provided.
(XLSX)
